# Risk factors associated with asthma, atopic dermatitis and rhinoconjunctivitis in a rural Senegalese cohort

**DOI:** 10.1186/s13223-015-0090-0

**Published:** 2015-08-25

**Authors:** Magali Herrant, Cheikh Loucoubar, Sabah Boufkhed, Hubert Bassène, Fatoumata Diene Sarr, Laurence Baril, Odile Mercereau-Puijalon, Salaheddine Mécheri, Anavaj Sakuntabhai, Richard Paul

**Affiliations:** Institut Pasteur, Unité de la Génétique Fonctionnelle des Maladies Infectieuses, 28 rue du Docteur Roux, 75724 Paris Cedex 15, France; Centre National de la Recherche Scientifique, Unité de Recherche Associée 3012, 75724 Paris Cedex 15, France; Institut Pasteur de Dakar, Unité d’Epidémiologie des Maladies Infectieuses, Dakar, Senegal; Institut Pasteur de Dakar, Group of Biostatistics, Bioinformatics and Modeling, Dakar, Senegal; Institut de Recherche pour le Développement (IRD), Unité de Recherche sur les Maladies Infectieuses et Tropicales Emergentes, URMITE CNRS-IRD 198 UMR 6236, Dakar, Senegal; Institut Pasteur, Unité d’Immunologie Moléculaire des Parasites, 75724 Paris Cedex 15, France; Institut Pasteur, Unité de Biologie des Interactions Hôte Parasite, 28 rue du Docteur Roux, 75724 Paris Cedex 15, France; Centre National de la Recherche Scientifique, Unité de Recherche Associée 2581, 75724 Paris Cedex 15, France

## Abstract

**Background:**

The World Allergy Organization estimates that 40 % of the world’s population is affected by allergic diseases. The International Study of Asthma and Allergies in Childhood has completed Phase III and it has now become clear that these diseases have increased in developing countries, especially Africa, where prevalence rates were formerly low. Despite an increase in studies in Africa, few sub-Saharan West African countries are represented; the focus has remained on urban populations and little attention has been paid to rural sub-Saharan Africa.

**Methods:**

We performed an allergy survey in a birth cohort of children aged less than 15 years in rural Senegal and implemented an ISAAC questionnaire. We carried out a complete blood count and serological analyses for IgE levels against common allergens and mosquito saliva.

**Results:**

The prevalence rates of asthma, rhinoconjunctivitis (RC) and atopic dermatitis (AD) were 12.8, 12.5 and 12.2 % respectively. Specific IgE (sIgE) levels against mosquito spp. salivary gland antigens were significantly associated with AD; sIgE levels against selected true grasses (Poaceae) were significantly associated with RC. sIgE levels against house dust mite spp. were not associated with asthma, but were significantly correlated with mosquito IgE levels. Such cross-reactivity may blur the association between HDM sIgE and asthma. Consumption of seafood, storing whey cream, using plant fibre bedding and presence of carpet were significantly associated with increased risk of RC. The association of seafood may be the result of histamine intoxication from molluscs prepared by putrefaction. Cat presence and dog contact were associated with increased risk of asthma. Cow contact was associated with increased risk of AD.

**Conclusions:**

Our allergy study in rural West Africa revealed lower prevalence rates than the majority of African urban settings. Although several associated known risk factors were identified, there were associations specific to the region. The identification of probable artefactual dietary phenomena is a challenge for robust diagnosis of allergic disease. The association AD with mosquito saliva, a common allergen in rural settings, warrants specific attention. Further studies in rural Africa are needed to address the aetiology of allergy in a non-urban environment.

**Electronic supplementary material:**

The online version of this article (doi:10.1186/s13223-015-0090-0) contains supplementary material, which is available to authorized users.

## Background

The World Allergy Organization estimates that 40 % of the world’s population is affected by allergic diseases [1]. The International Study of Asthma and Allergies in Childhood (ISAAC) is a global consortium that was established in 1991 to investigate asthma, rhinitis and eczema in children from more than 100 countries [2]. The ISAAC diagnostic criteria have been shown to be reproducible, adequate and able to discriminate children with allergic diseases in different areas of the world [2]. With completion of Phase III, it has now become clear that these allergic diseases have increased in developing countries, especially Africa, where prevalence rates were formerly low [3]. Despite an increase in the number of consortial centres in Africa in Phase III, however, few sub-Saharan West African countries are represented and the focus has remained on urban populations [4]. The urban focus enables a sufficient recruitment sample size for global comparisons of prevalence rates and implicitly targets the population most likely to have allergic disease [5]. This, however, introduces a bias and can not provide an overall picture of variation in prevalence rates.

In addition to assessing global trends, the ISAAC consortium generated the framework within which the aetiology of these diseases could be addressed. Several ecological studies aimed at identifying environmental risk factors associated with these allergic diseases have been performed, enabling population level analysis of risk [6]. These studies have notably found several factors that individually have small effects on prevalence, including Gross National Product, diet, antibiotic use, climate and pollution, amongst others [6]. At the other end of the spectrum are more detailed individual based analyses that can assess local scale factors associated with risk, which may be more specific to local populations.

To date, with the exception of Southern Africa [7], few risk analyses on allergic disease have been performed in rural sub-Saharan Africa and none have implemented a generalised ISAAC methodology [8–12]. We have previously reported on the impact of asthma and atopic dermatitis on the acquisition of clinical immunity to malaria in a birth cohort in Senegal [13]. Here we present the analysis of risk factors associated with asthma, rhinoconjunctivitis and atopic dermatitis, established through implementation of a locally adapted ISAAC questionnaire in this population. In addition we performed serological analyses for specific IgE against common allergens, including mosquito saliva, house dust mite, and Poaceae spp. (taxon including true grasses) and we measured eosinophil density to determine eosinophilia status.

## Methods

Our study cohort consists of two adjacent villages located in the district of Fatick, Dielmo and Ndiop, that have participated in a malaria research program since the 1990s, in which active and passive surveillance is carried out to identify clinical malaria episodes [14]. The two villages are 5 km apart, situated in the dry Sahel in a very dry, rural environment typical of the region. Specifically there is no running water and water must be drawn from several wells distributed throughout the villages. There are no power lines bringing electricity to the houses and people use a variety of systems to generate light; these include candles, car batteries, kerosene lamps and solar panels. Cooking is performed in Malagasy portable iron ovens, on portable gas burners or on open fires using wood and charcoal. Heating, when needed, is achieved via the ovens or open fires. There is no refrigeration. Household furnishing is basic and a variety of synthetic, plant (straw, dried plant matter) or animal products (feathers) are used in the mattress composition. The villagers maintain livestock, notably cows, chickens and goats from which they generate a variety of dairy products that are processed to enable at least short-term storage without the need for refrigeration. Whey cream is one example, where cream is skimmed off the whey part of milk and used to flavour foods. During the rainy season, the villagers grow and harvest a variety of staple foods, including millet, wheat, sorghum, maize, manioc and nuts and additionally consume couscous and rice purchased locally. Animal protein for consumption includes eggs, occasionally fish and mutton. In addition, one regular dietary component is “Yeet”, a mollusc processed and used to flavour food. The mollusc is tenderized by putrefaction (microbial decomposition of organic matter). Finally, because of the burden of malaria, insecticide-treated bednets are present throughout the villages.

We conducted a cross-sectional survey to estimate the prevalence of symptoms related to allergic diseases among 321 children aged from 1 month to 15 years who were born during the malaria research program and thus for whom complete medical records exist. There were 16 children less than 1 year of age, 107 between 1 and 5 years of age, 105 between 5 and 10 years of age and 93 between 10 and 15 years of age. The children were recruited as follows: 255 children were unique to a household, 46 were recruited as pairs, 12 as triples and eight as quadruples from the same household. The allergy study was approved by the Senegalese National Ethics committee (2009/No 46).

After presenting information about the procedures and the purpose of the study, written informed consent was obtained from parents or guardians of children either by signature or by thumbprint on a voluntary consent form written in both French and Wolof, the main local language. Consent was obtained in the presence of an independent witness (the school director).

### Allergic diseases and atopic status

The standardized ISAAC questionnaire originally written in English was translated into French in compliance with ISAAC guidelines [15], adapting it to the usual local customs following advice from local clinicians and paediatric allergologists (Additional file 1). The adequacy and reliability of the translated questionnaire had been previously confirmed by a pilot study on 30 randomly selected children from the same community. The questionnaire was completed by specially trained health workers during an oral interview conducted in Wolof with children and their mothers or guardians. A description of the ISAAC variables measured is given in the Additional file 2.

To assess the prevalence of allergic diseases in children, we used the positive and negative predictive values of the ISAAC questionnaire diagnosis criteria developed for subtropical countries [16]. Each question was scored according to the medical diagnosis of paediatricians and paediatric allergologists. Positive or negative answers were thus graded on the basis of symptom sensitivity, specificity, frequency, location or early onset. For each allergic disease, three categories of symptom severity, *severe*, *moderate*, and *none*, were defined (see Additional file 2) [13].

The inter-relationships between variables reflecting the severity of symptoms of the three allergic diseases were used to identify children at high risk of atopy. The *high probability* group was defined by the prevalence of at least one of any *severe* symptoms or two of any *moderate* symptoms. The *probable* group was defined as those with *moderate* symptoms from one of the three allergic diseases and remaining children were classified in the *unlikely* group.

### Laboratory analyses

An intestinal helminth survey was carried out for 194 of the participating individuals for whom we were able to obtain stool samples. Diagnosis was performed by stool examination for parasites and eggs by microscope and by the more sensitive Kato-Katz technique in order to search for the presence of *Ascarislumbricoides*, *Ancylostoma duodenale* and *Necator americanus*, *Trichuris trichiuria*, *Schistosomamansoni* and *Strongyloidesstercoralis* [17, 18]. Examination for *Enterobius vermicularis* was performed by the anal scotch-test. An anti-helminthic treatment was proposed for all infested individuals. A blood sample was obtained from 168 individuals for a complete blood count and immunoglobulin E titration. Specific IgE levels against mosquito spp. salivary gland extract, house dust mite spp. and a mix of pollen allergens (Additional file 2) were measured by ELISA as previously described [19].

### Statistical analysis

Statistical analyses were performed using Genstat ver. 15 (VSN Ltd) [20]. An association of variables on the risk of allergy was analysed by logistic regression; allergy classes were reduced to two levels, *severe* or *moderate* vs. *none*. In order to reduce the number of tests performed, we first analysed using groups of explanatory variables (by type, as shown in the Additional file 2) in multivariate analyses and then selected those variables with P < 0.2 for the final multivariate analysis. To address age-specific associations, we then performed a multivariate analysis for each of two age groups (0–7 years old vs. 8–15 years old) using those variables found to be significantly associated in the global analysis. The association of allergy class with IgE levels and eosinophilia count was analysed by linear regression using log transformed data.

## Results and discussion

Of the 443 eligible children aged less than 15 years, 321 participated in the cross-sectional survey to assess the prevalence of symptoms of allergic diseases. All eligible children present at the time of the survey were included; no explicit refusal to participate was recorded. The study cohort was aged from 1 month to 14.9 years with a median interquartile range (IQR) age of 6.3 (3.1–10.7) years. The sex-ratio (male/female) was 0.91.

The prevalence of moderate or severe asthma symptoms was, respectively 2.2 % [95 % confidence interval (CI) 0.9–4.4] and 10.6 % (95 % CI 7.4–14.5). The prevalence of moderate or severe allergic rhinoconjunctivitis symptoms was, respectively 4.4 % (95 % CI 2.4–7.2) and 8.1 % (95 % CI 5.4–11.6). The prevalence of moderate or severe atopic dermatitis symptoms was, respectively 7.8 % (95 % CI 5.1–11.3) and 4.4 % (95 % CI 2.4–7.2). There were no significant effects of age or gender on any allergic disease. On the basis of symptom severity, an atopic tendency was estimated to be unlikely for 67.0 % (95 % CI 61.5–72.1), probable for 11.5 % (95 % CI 8.2–15.5) and highly probable for 21.5 % (95 % CI 17.1–26.4) of the 321 children less than 15 years old. The higher rates of severe Asthma or severe RC, compared to moderate symptoms of these conditions, are surprising, but may be the result of the subjective nature of differentiation between moderate and severe levels of symptoms.

Combining moderate and severe categories, the overall prevalence rates of Asthma, RC and AD were 12.8, 12.5 and 12.2 %, respectively (Table 1). The prevalence rates of multiple allergies were 2.5 % for Asthma + RC and less than 1 % for AD + RC and Asthma + AD; only two individuals had all three allergic diseases. In West African countries that participated in the ISAAC consortium, Ivory Coast and Guinea Conakry, prevalence rates were much higher, ranging from 18 to 28 % [4]. The prevalence rates in our study are more comparable to those observed in Yaoundé and Kinshasa for the same age groups (Wheeze 7 %, RC 9–12 % and AD 7–11 %) [4]; this similarity is surprising given the contrasting rural and densely populated urban nature of the sites.

**Table 1:** Study participants by age, gender and clinical symptoms of allergy and atopic tendency

Gender	Age group (years)						Total	
	(0–5)		(5–10)		(10–15)			
	M	F	M	F	M	F	M	F
Total	60	63	56	49	37	56	153	168
Asthma								
No	51	54	50	44	28	53	129	151
Yes	9	9	6	5	9	3	24	17
Rhinoconjunctivitis								
No	51	53	53	41	32	51	136	145
Yes	9	10	3	8	5	5	17	23
Atopic dermatitis								
No	53	53	44	45	34	53	131	151
Yes	7	10	12	4	3	3	22	17
Atopic tendency								
Unlikely	40	36	38	35	21	45	99	116
Probable	1	9	11	5	4	7	16	21
Highly probable	19	18	7	9	12	4	38	31

The prevalence of helminths was 3.6 % (95 % CI 1.5–7.3; n = 7 infested out of 194 children tested). Three children were infested with *Ancylostoma*, one with *Strongyloides*, two with *Enterobius* and one with *Trichuris*.

**Fig. 1:**
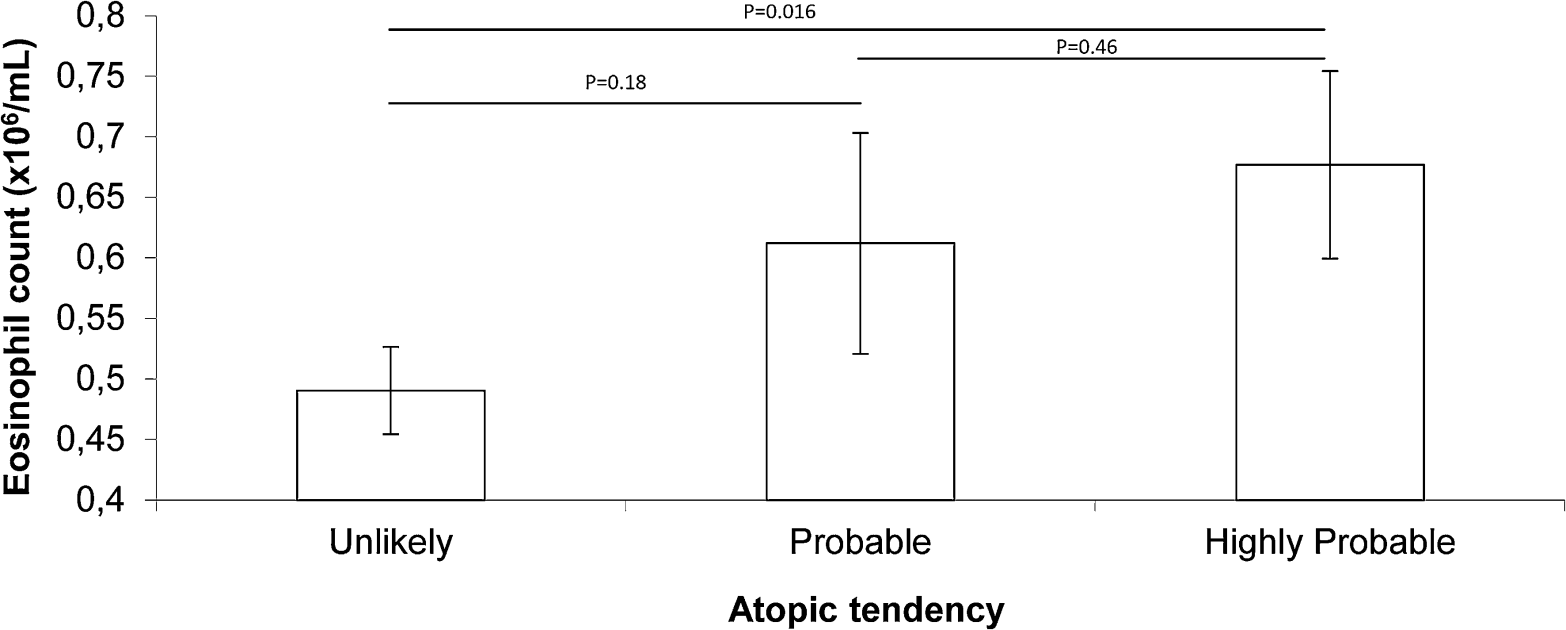
Eosinophil count according to atopic tendency. Shown are means (±SEM) and P values

The eosinophil count was found to decrease with age (F_1,164_ = 35.5, P < 0.001) but increase with the atopic tendency groups (probable and highly probable vs. no tendency) (F_1,164_ = 6.63, P = 0.025) (Fig. 1). The presence of helminth infections had no effect on the eosinophil count (F_1,163_ = 0.63, P = 0.43) (absence: mean 0.584 × 10^6^/mL ± SEM 0.046 n = 163, vs. presence: mean 0.406 × 10^6^/mL ± SEM 0.11 n = 5).

**Fig. 2:**
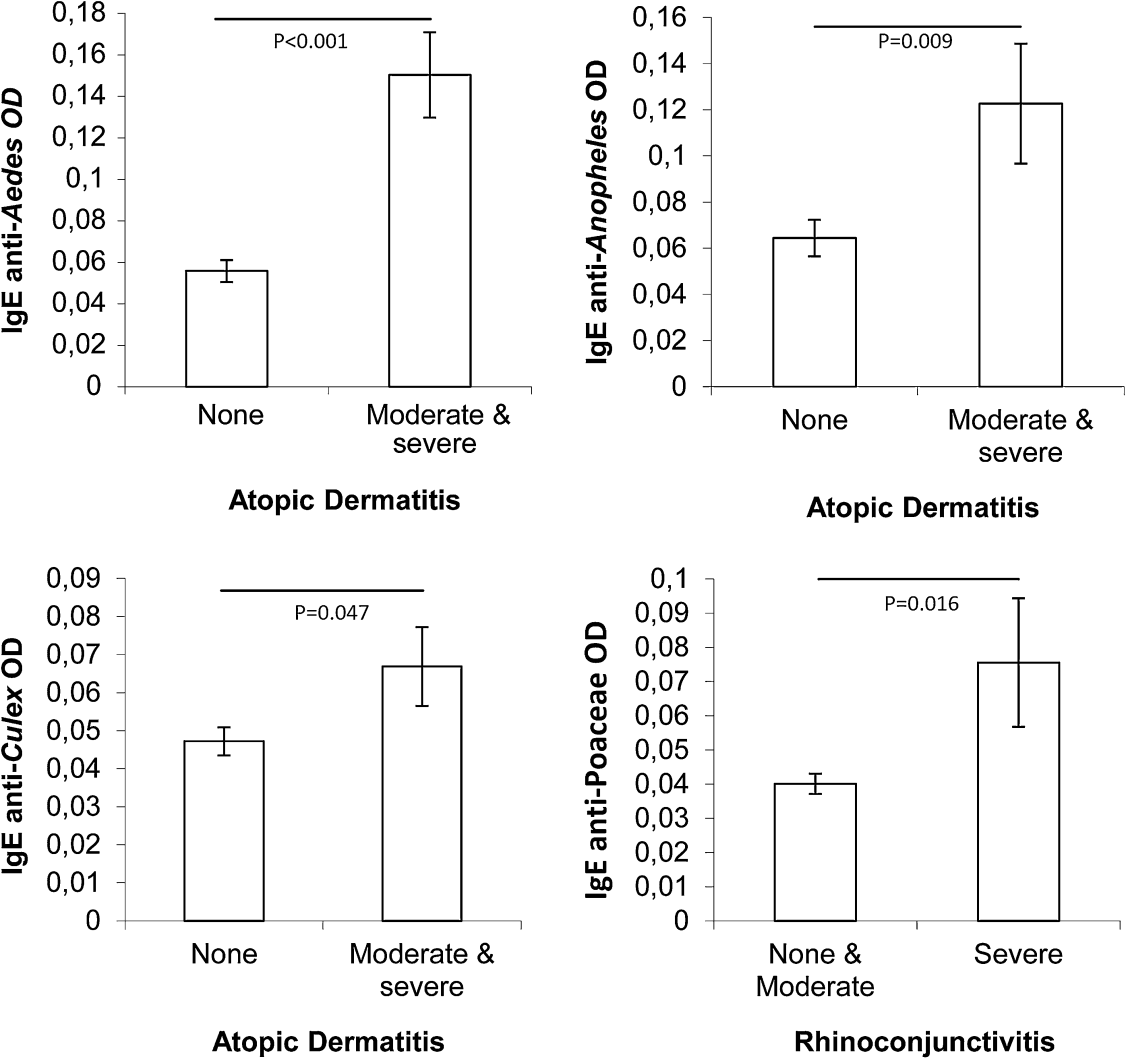
Specific IgE levels against selected mosquito and grass allergens according to severity of allergy symptoms. Allergens are: salivary gland extracts of three mosquito species, *Aedes aegypti*, *Anopheles gambiae* s.s. and *Culex quinquefasciatus* and a mix of several *Poaceae*. Shown are means (±SEM) and P values

Individuals with moderate or severe symptoms of AD had significantly higher specific IgE levels against all three mosquito spp. salivary gland extract: *Aedes aegypti* (F_1,155_ = 30.3, P < 0.001), *Anopheles gambiae* salivary gland extract (F_1,156_ = 7.00, P = 0.009) and *Culex quinquefasciatus* (F_1,156_ = 4.01, P = 0.047) (Fig. 2). Individuals with severe symptoms of RC had significantly higher IgE levels against the tested Poaceae spp. (F_1,156_ = 5.93, P = 0.016). There was no association of asthma with IgE levels against the house dust mite spp. tested (*D. farinae* P = 0.84 and *D. pteronyssinus* P = 0.68; Additional file 2: Figure). IgE values were strongly correlated for the three mosquito genera and interestingly also for mite IgE with *Anopheles* (Table 2). Poaceae spp. IgE levels were not correlated with other specific IgE levels.

**Table 2:** Correlations among IgE levels and 2-sided tests for differences from zero

*Aedes*	–				
*Anopheles*	0.622***	–			
*Culex*	0.607***	0.566***	–		
*D. farinae*	0.223	0.360*	0.131	–	
*D. pteronyssinus*	0.249	0.381**	0.140	0.599***	–
Poaceae	0.187	0.070	−0.049	−0.034	−0.164
	*Aedes*	*Anopheles*	*Culex*	*D. farinae*	*D. pteronyssinus*

* P < 0.05; ** P < 0.01; *** P < 0.001

The significant associations of mosquito saliva sIgE levels with AD and Poaceae spp. IgE levels with RC lend support to the clinical diagnosis of these two diseases, as does the association of eosinophilia with atopy. The lack of association of mite IgE levels with asthma has been observed previously [21]; the significant positive correlation with mosquito IgE suggests that not only do these arthropods share common antigens [22], but also that the cross-reactivity may blur any association between asthma and mite IgE [23]. The significance of mosquito saliva as an allergen in rural tropical countries needs to be taken into account when assessing risk factors for AD.

### ISAAC analysis

#### Asthma

The presence of a cat in the house or direct contact with dogs was associated with an increased risk of asthma [Cat: adjusted odds ratio (ORa) 5.3, 95 % CI 1.49–18.8, P = 0.01; Dog: ORa 2.28, 95 % CI 1.26–4.11, P = 0.006] (Table 3A). By contrast, presence of chickens and use of a synthetic fibre mattress were associated with a decreased risk of asthma (chicken: ORa 0.18, 95 % CI 0.05–0.64, P = 0.008; synthetic bedding: ORa 0.43, 95 % CI 0.19–0.96, P = 0.039).

**Table 3:** Distribution of study participants by exposure to risk factors for allergy-related conditions

A. Asthma	Asthma status						
	No		Yes		Total		P-value
	N	%	N	%	N	%	
Total	280	87.2	41	12.8	321	100	
Risk factor							
Cat presence							0.01
No	53	19.0	3	7.3	56	17.5	
Yes	226	81.0	38	92.7	264	82.5	
Dog contact							0.006
No	172	61.6	16	40.0	188	58.9	
Yes	107	38.4	24	60.0	131	41.1	
Chicken presence							0.008
No	18	6.4	4	9.8	22	6.9	
Yes	262	93.6	37	90.2	299	93.1	
Child’s mattress composition							0.04
Other	26	9.3	7	17.1	33	10.3	
Synthetic	254	90.7	34	82.9	288	89.7	

B. Atopic dermatitis	AD status						
	No		Yes		Total		P-value
	N	%	N	%	N	%	
Total	282	87.9	39	12.1	321	100	
Risk factor							
Cat presence							0.048
No	46	16.4	10	25.6	56	17.5	
Yes	235	83.6	29	74.4	264	82.5	
Cow presence							0.006
No	189	67.0	19	48.7	208	64.8	
Yes	93	33.0	20	51.3	113	35.2	

C. Rhinoconjunctivitis	RC status						
	No		Yes		Total		P-value
	N	%	N	%	N	%	
Total	281	87.5	40	12.5	321	100	
Risk factor							
History of helminth infestation							0.005
No	218	77.9	25	62.5	243	75.9	
Yes	62	22.1	15	37.5	77	24.1	
Chicken contact							0.007
No	53	18.9	2	5.0	55	17.1	
Yes	228	81.1	38	95.0	266	82.9	
Child’s mattress composition							<0.001
Straw and other dry plant matter	265	94.3	34	85.0	299	93.1	
Other	16	5.7	6	15.0	22	6.9	
Cooker							0.033
No	156	55.5	26	65.0	182	56.7	
Yes	125	44.5	14	35.0	139	43.3	
Solar							0.014
No	46	16.4	9	22.5	55	17.1	
Yes	235	83.6	31	77.5	266	82.9	
Storing whey cream							<0.001
No	254	90.4	26	65.0	280	87.2	
Yes	27	9.6	14	35.0	41	12.8	
Stock millet							0.042
No	148	52.7	24	60.0	172	53.6	
Yes	133	47.3	16	40.0	149	46.4	
Rug							<0.001
No	179	63.7	10	25.0	189	58.9	
Yes	102	36.3	30	75.0	132	41.1	
Consumption of sea food							
Never	71	25.3	2	5.0	73	22.7	
<Once/week	149	53.0	17	42.5	166	51.7	0.01
1–2 times/week	47	16.7	15	37.5	62	19.3	<0.001
≥Once/day	14	5.0	6	15.0	20	6.2	0.002
Weight							<0.001
Mean + SD Weight (n)	20.6 + 10.3 (280)		18.3 + 10.2 (40)				

D. Atopy	Atopy status						P-value
	No		Yes		Total		
	N	%	N	%	N	%	
Total	252	78.5	69	21.5	321	100	
Risk factor							
Storing whey cream							0.021
No	222	89.5	53	77.9	275	87.0	
Yes	26	10.5	15	22.1	41	13.0	

For each risk factor, shown are the number and percentages of participants with/without the risk factor for each allergic status (No/Yes). Also shown are the corresponding P values in the final analyses. A. Asthma, B. Atopic dermatitis, C. Rhinoconjunctivitis, D. Atopy

#### Atopic dermatitis (AD)

Contact with cows was associated with an increased risk of AD (ORa 2.29, 95 % CI 1.27–4.11, P = 0.006) (Table 3B). By contrast, presence of a cat in the house was associated with a decreased risk of AD (ORa 0.5, 95 % CI 0.25–0.99, P = 0.048).

#### Rhinoconjunctivitis (RC)

Numerous factors were associated with increased or decreased risk of RC (Table 3C):

1. Decreased risk: possessing a cooker (ORa 0.50, 95 % CI 0.26–0.95, P = 0.033); solar panels (ORa 0.39, 95 % CI 0.18–0.83, P = 0.014); storing millet in the sleeping area (ORa 0.94, 95 % CI 0.27–0.98, P = 0.042); weight (ORa for 0.1 kg increase 0.94, 95 % CI 0.91–0.97, P < 0.001).
2. Increased risk: a history of helminth infestation (ORa 2.54, 95 % CI 1.33–4.84, P = 0.005); exclusively plant fibre bedding (ORa 12.79, 95 % CI 4.53–36.07, P < 0.001); storing whey cream in the sleeping area (ORa 13.64, 95 % CI 5.86–31.75, P < 0.001); contact with chickens (ORa 6.14, 95 % CI 1.65–22.87, P = 0.007); presence of a rug (ORa 13.35, 95 % CI 5.99–29.77, P < 0.001); consumption of seafood (less than once/week, ORa 5.02, 95 % CI 1.47–17.07, P = 0.01; 1–2 times/week, ORa 9.20, 95 % CI 2.62–32.22, P < 0.001; once a day, ORa 16.99, 95 % CI 3.72–77.66 P < 0.001, P = 0.002).

#### Atopy

An increased risk of atopy was associated with storing whey cream (OR 2.41, 95 % CI 1.14–5.08, P = 0.021) (Table 3D).

### Correction for multiple testing

Eight multivariate analyses were carried out for the initial identification of potentially important variables prior to the final analysis and this for each allergy category plus the predicted atopy status. This yielded 36 statistical tests and a Bonferroni corrected *P* value threshold of P = 0.00139. There were no risk factors associated with asthma, atopic dermatitis or atopy at this threshold. For rhinoconjunctivitis, however, a higher body weight decreased risk. Exclusively plant fibre bedding, storing whey cream, presence of a carpet/rug and regular consumption of seafood were associated with increased risk of rhinoconjunctivitis.

### Factors in young (0–7 years, N = 168) vs. older (8–15 years, N = 153) children

Risk factors might be expected to differentially impact upon allergy in children of differing ages and the association of the significant variables found in the global analysis was tested for young and older aged children. Dividing the children into young and older children did not reveal any substantial differences in the direction of the association, although the significance of the association was affected. In general the associations were stronger in younger children. Dog contact was associated with increased risk of asthma only in the younger children (ORa 3.50, 95 % CI 1.35–9.09, P = 0.01) (Table 4). In the younger children, an increased risk of RC was associated with history of helminth infection (ORa 3.91, 95 % CI 1.29–11.81, P = 0.016) and consumption of seafood 1–2 times per week (ORa 9.27, 95 % CI 1.52–56.66, P = 0.016) or more than that (ORa 18.75, 95 % CI 1.99–176.50, P = 0.010) and storing whey cream (ORa 13.20, 95 % CI 3.13–55.77, P < 0.001). The presence of solar panels was associated with decreased risk of rhinoconjunctivitis (ORa 0.18, 95 % CI 0.05–0.65, P = 0.008). Cow contact was associated with AD only in older children (ORa 3.67, 95 % CI 1.08–12.47, P = 0.037). Storing whey cream was associated with increased risk of atopy in children 8–15 years old (ORa 3.84, 95 % CI 1.51–9.77, P = 0.005). The presence of carpets/rug was associated with an increased risk of RC in both age groups (age 1–7: ORa 6.49, 95 % CI 1.88–22.45, P = 0.003; age 8–15: ORa 10.50, 95 % CI 2.07–53.30, P = 0.005).

**Table 4:** Differential association of significant variables with allergy-related conditions by age group

Age group (years)	0–7 (N = 168)			8–15 (N = 153)		
	ORa	95 % CI	P value	ORa	95 % CI	P-value
Asthma						
Cat presence	3.60	0.62–20.91	0.153	14.80	0.84–261.1	0.066
Dog contact	***3.50***	***1.35***–***9.09***	***0.01***	1.35	0.48–3.79	0.566
Chicken presence	0.24	0.03–1.69	0.153	0.10	0.01–1.19	0.068
Synthetic bedding	0.34	0.09–1.28	0.11	0.53	0.13–2.14	0.369
AD						
Cow contact	2.19	0.87–5.54	0.098	***3.67***	***1.08***–***12.47***	***0.037***
Cat presence	0.38	0.14–1.03	0.058	0.67	0.17–2.70	0.573
RC						
History of helminths	***3.91***	***1.29***–***11.81***	***0.016***	1.27	0.30–5.31	0.744
Rug/carpet	***6.49***	***1.88***–***22.45***	***0.003***	***10.50***	***2.07***–***53.30***	***0.005***
Solar panels	***0.18***	***0.05***–***0.65***	***0.008***	1.66	0.18–15.34	0.657
Seafood < once a week	5.49	0.97–30.95	0.054	2458.00	*10* ^−*15*^–*10* ^*21*^	0.71
Seafood 1–2 per week	***9.27***	***1.52***–***56.66***	***0.016***	3722.00	*10* ^−*15*^–*10* ^*21*^	0.695
Seafood once a day	***18.75***	***1.99***–***176.5***	***0.01***	2664.00	*10* ^−*15*^–*10* ^*21*^	0.707
Stocking whey cream	***13.20***	***3.13***–***55.77***	***<.001***	4.49	0.99–20.47	0.052
Atopy						
Stocking whey cream	1.82	0.69–4.80	0.226	***3.84***	***1.51***–***9.77***	***0.005***

*ORa* adjusted Odds Ratio in the multivariate analysis

Bold italics indicate significant associations with a threshold of P < 0.05

Food allergies, namely the consumption of seafood and the storing of whey cream had a significant impact on risk of RC. Whey cream is cream skimmed from whey and has a stronger flavour than cream skimmed from milk and is frequently used to flavour foods. The strong associations observed with this dairy product may reflect higher exposure of family members to cattle. Additionally, it may be related to increased consumption of trans-fatty acids, a risk factor previously identified in the ISAAC consortium surveys [6]. A major component of seafood in this population is “Yeet”, a mollusc processed and used to flavour food; prawns and mangrove oysters are consumed but to a much lesser extent. The mollusc is tenderized by putrefaction (microbial decomposition of organic matter), during which time there is increased histamine production by the microbial flora [24]. The extent to which the increased risk of RC is due to the immediate effects of histamine contamination rather than a sustained allergic state, is not clear. The most significant factor associated with increased risk of RC was the presence of carpet/rugs.

Indoor air quality is known to impact upon the risk of allergic diseases; mould, dust and pets have been associated with asthma, RC and AD [25, 26]. Here, the positive association of the presence of rugs and use of plant fibre bedding with increased risk of RC is consistent with their negative effects on air quality. Likewise, the association of decreased risk of RC with the presence of solar panels likely reflects a reduced use of kerosene lamps and improved indoor air quality.

In this rural environment, where animals wander at will among the concessions (family level collection of huts/constructions with a perimeter fence), the low level of variation in animal contact and presence among individuals would be expected to reduce any impact of animal “effects” on allergic disease. However, small but significant effects were identified and were largely consistent with previous observations. Notably, exposure to cats and contact with dogs increased the risk of asthma, as has been previously found [26], whereas exposure to poultry in early life reduced risk; this latter is consistent with the hygiene hypothesis [27]. By contrast, cow contact increased AD and was surprisingly consistent across age groups. An ISAAC global study found that in non-affluent countries early-life exposure to farm animals was associated with increased symptoms of asthma, RC and eczema in 6- to 7-year-old children [28]. Cow hair and dander can result in contact dermatitis [29]; younger children, however, may develop atopic dermatitis because of cow milk allergy [30].

In more affluent countries, it has long been recognised that farmer’s children have reduced incidence of allergic diseases and especially asthma [31], which has been ascribed to the high microbial diversity in such environments [32]. Indeed, recent studies have increasingly highlighted the importance of microbial diversity in contrast to single microbial markers in determining the risk of asthma [33]. In addition to the fungal and bacterial community, respiratory viral infections during early life have been shown to exacerbate the risk of allergic disease and especially asthma [34, 35]. The viral infections have been hypothesised to induce a Th2 response that then predisposes the individual to an IgE mediated immune response in the presence of the allergen [36]. In our study site, malaria is the major infectious disease and we have previously shown that allergic diseases, notably asthma and AD, negatively impacted upon the immune response to malaria, potentially causing an imbalance in the Th1/Th2 response [13]. How the community of infectious diseases impacts upon allergic diseases and vice versa needs to be considered in risk factor analysis for allergic diseases.

Despite the relatively small sample size and the small level of variation in many of the variables measured, several risk factors were identified that are consistent with what has been previously observed. Notable novelties of this study were the identification of an association of AD with mosquito saliva sIgE and an association of RC with consumption of seafood. Mosquito bites are a wide-spread problem in rural sub-Saharan Africa and the extent to which they pose a risk for AD needs to be addressed. The observed risk associated with seafood consumption may well be a problem of food histamine intoxication, which could be remedied with improved food hygiene. The protective effect of some species of helminth infections has been extensively discussed, especially in the context of the hygiene hypothesis [37]. The best evidence for protection seems to come from infection by *Schistosoma* spp. [12]. *Schistosoma* spp. do not occur in this part of the Sahel. The close medical follow-up of our cohorts, albeit focused on malaria, have led to extensive anti-helminth treatment. Thus, in contrast to the majority of rural populations, helminth prevalence was too low in our cohorts to be able to make any assessment of their impact on allergic diseases.

## Conclusions

Our allergy study in rural West Africa revealed lower prevalence rates of allergic diseases than the majority of African urban settings but which were nevertheless not insignificant. There were several risk factors associated with allergy and which were to some extent age-dependent. Notably the presence of a rug in the dwelling increased risk of RC 13-fold. Food-related risk factors, notably seafood and storage of whey cream increased RC. These are likely to be specific to the region. Both associations may, however, be artefacts of food hygiene. Such very specific phenomena are a challenge for robust diagnosis of allergic disease. There were no strongly associated risk factors for Asthma and AD, although the association of mosquito bites with AD requires further investigation. Further studies in rural Africa are needed to address the aetiology of allergy in a non-urban environment, not least to provide a baseline for comparison of the effect of urbanisation.

## Additional files

Additional file 1. ISAAC questionnaire in English Additional file 2. Allergy classification criteria, description of ISAAC variables and details of allergens and immunoglobulin E titres

## Abbreviations

ISAAC: International Study of Asthma and Allergies in Childhood
TELC: tropical endemic limboconjunctivitis
BMI: body mass index
AD: atopic dermatitis
RC: rhinoconjunctivitis
CI: confidence interval
