# Supplementary material for: Risk factors associated with asthma, atopic dermatitis and rhinoconjunctivitis in a rural Senegalese cohort
Source: Allergy Asthma Clin Immunol. 2015 Aug 25;11(1):24. doi: 10.1186/s13223-015-0090-0 (PMC4547418; doi:10.1186/s13223-015-0090-0)
Supplement: Additional file 1. — ISAAC questionnaire in English [file 13223_2015_90_MOESM1_ESM.doc]

| ***Place of study***  *Technician X*  *Technician X*  *Technician X*  🕿 + | ***Research Institute responsible***  *Name of Institute*  *Principle investigator*  *Project manager*  🕿 + |
| --- | --- |

| **IDENTIFICATION**  Date of questionnaire : |__|__| |__|__| |__|__|__|__| dd/*mm/yyyy*  Name of investigator :……………………………………………………………………………  Name of study supervisor :……………………………………………………………  ***Child :***  **First and last name of child :**  **Identification code of child :**  **Date of birth :**  **Sex****:**  **Village/town :**    **Identification code of Questionnaire :**  **Identification code of father**:  **Identification code of mother :**  **Weight : |__|__|,|__| (kg)**  **Height : |__|__|__|,|__| (cm)**  **Mid Upper Arm Circumference: |__|__| (cm)**  ***Person questioned :***  Last name of person questioned :……………………………………………………………………..……  First name of person questioned :…………………………………………………………………….…  Relationship to child : Mother _1_ Father _2_ Brother/Sister _3_ Grand-parents _4_  Other _5_  If other, define : …………………………...........................................................  **FACTORS PREDISPOSING ATOPY**  ***First days of life :*** Consultation of health records of child and maternity records of mother   1. How much did your child weigh at birth ?   <1500 g _1_ [2500-3500[ g _4_  [1500-2000[ g _2_ ≥ 3500 g _5_  [2000-2500[ g _3_ Record not found/NSP_9_   1. Until what age did your child breastfeed (**exclusively or mixed**) ?   *Corresponds to the age of weaning of child*  < 6 months _1_ 6 – 12 mths _2_ 12 – 24 mths _3 >_ 24 mths _4_  NSP _9_ | **Validation zone**  |__|__| |__|__| |__|__|__|__| DTEQUE  |__|__| |__|__| IDENF  |__|__| |__|__| |__|__|__|__| DOB  |__| SEXE  |__| VILLAGE  |__| |__|__| |__| |__|__||__|__| IDQUES  |__|__| |__|__| IDFA  |__|__| |__|__| IDMO  |__|__|,|__| (Kg) WEIGHT  |__|__|__|,|__| (cm) HEIGHT  |__|__| (cm) MUAC  ………………………………… NAMEPQ  ………………………………… LASTNAMEPQ  |__| RELCHILD  ………………………………… OTHERREL  |__| BIRTHWEIGH  |__| AGEWEAN |
| --- | --- |
| 1. Until what age did your child breastfeed **exclusively** without ever taking other aliments (fruits, vegetables, rice, meat, fish, etc.) or liquids (powdered milk, cow or goats milk, fruit juice, water, etc.) ?   < 6 months _1_ 6 – 12 mths _2_ 12 – 24 mths _3_ NSP _9_  ***Illness and vaccination :*** Consultation of health records of child   1. Has your child enfant had the following illnesses?   Malaria : _0_ No _1_ Yes _9_ NSP  Tuberculosis treated : _0_ No _1_ Yes _9_ NSP  Helminths (oxyures, ascaris, taenia, etc.) : _0_ No _1_ Yes _9_ NSP  Amoeba : _0_ No _1_ Yes _9_ NSP  Measles : _0_ No _1_ Yes _9_ NSP   1. Against what illnesses is you child vaccinated?   Yellow fever : _0_ No _1_ Yes _9_ NSP  Hepatitis B : _0_ No _1_ Yes _9_ NSP  Measles : _0_ No _1_ Yes _9_ NSP  Mumps : _0_ No _1_ Yes _9_ NSP  Rubella : _0_ No _1_ Yes _9_ NSP  Tuberculosis/BCG : _0_ No _1_ Yes _9_ NSP  Diphtheria/Tetanus/Pertussis/Poliomyelitis : _0_ No _1_ Yes _9_ NSP  Typhoid : _0_ No _1_ Yes _9_ NSP  Meningitis : _0_ No _1_ Yes _9_ NSP  Haemophilus influenzae type B (HiB) : _0_ No _1_ Yes _9_ NSP  ***Habitation :***   1. Which of these animals / insects can be found in the **rooms** where your child lives (today and/or during his first year of life) ?   Dogs in rooms today : _0_ No _1_ Yes _9_ NSP  Dogs in rooms 0-1yr : _0_ No _1_ Yes _9_ NSP  Cats in rooms today : _0_ No _1_ Yes _9_ NSP  Cats in rooms 0-1yr : _0_ No _1_ Yes _9_ NSP  Sheep in rooms today : _0_ No _1_ Yes _9_ NSP  Sheep in rooms 0-1yr : _0_ No _1_ Yes _9_ NSP  Goats in rooms today : _0_ No _1_ Yes _9_ NSP  Goats in rooms 0-1yr : _0_ No _1_ Yes _9_ NSP  Chicken, ducks in rooms today : _0_ No _1_ Yes _9_ NSP  Chicken, ducks in rooms 0-1yr : _0_ No _1_ Yes _9_ NSP  Rodents (rats, mice, etc.) in rooms today : _0_ No _1_ Yes _9_ NSP  Rodents (rats, mice, etc.) in rooms 0-1yr : _0_ No _1_ Yes _9_ NSP  Cockroaches in rooms today : _0_ No _1_ Yes _9_ NSP  Cockroaches in rooms 0-1yr : _0_ No _1_ Yes _9_ NSP  Other in rooms today : _0_ No _1_ Yes _9_ NSP  Other in rooms 0-1yr : _0_ No _1_ Yes _9_ NSP  If Others, define :……………………………………………   1. Which of these animals could be in **contact** with your child **at least once per week** (today and/or during his first year of life) ?   Contact with Dogs today : _0_ No _1_ Yes _9_ NSP  Contact with Dogs 0-1yr : _0_ No _1_ Yes _9_ NSP  Contact with Cats today : _0_ No _1_ Yes _9_ NSP  Contact with Cats 0-1yr : _0_ No _1_ Yes _9_ NSP  Contact with Sheep today : _0_ No _1_ Yes _9_ NSP  Contact with Sheep 0-1yr : _0_ No _1_ Yes _9_ NSP  Contact with Goats today : _0_ No _1_ Yes _9_ NSP  Contact with Goats 0-1yr : _0_ No _1_ Yes _9_ NSP  Contact with Chicken, Ducks today : _0_ No _1_ Yes _9_ NSP  Contact with Chicken, Ducks 0-1yr : _0_ No _1_ Yes _9_ NSP  Contact with donkeys, horses today : _0_ No _1_ Yes _9_ NSP  Contact with donkeys, horses 0-1yr : _0_ No _1_ Yes _9_ NSP  Contact with Cows, zébus today : _0_ No _1_ Yes _9_ NSP  Contact with Cows, zébus 0-1yr : _0_ No _1_ Yes _9_ NSP  Contact with Rodents (rats, mice, etc.) today : _0_ No _1_ Yes _9_ NSP  Contact with Rodents (rats, mice, etc.) 0-1yr : _0_ No _1_ Yes _9_ NSP  Contact with Other today : _0_ No _1_ Yes _9_ NSP  Contact with Other 0-1yr : _0_ No _1_ Yes _9_ NSP  If Others, define :……………………………………………   1. Which of these aliments are usually stocked in the rooms where your child lives ?   Millet kept in room : _0_ No _1_ Yes _9_ NSP  Sorghum kept in room : _0_ No _1_ Yes _9_ NSP  Maize kept in room : _0_ No _1_ Yes _9_ NSP  Rice kept in room : _0_ No _1_ Yes _9_ NSP  Wheat kept in room : _0_ No _1_ Yes _9_ NSP  Biscuits, pasta kept in room : _0_ No _1_ Yes _9_ NSP  Manioc (root, flour) kept in room : _0_ No _1_ Yes _9_ NSP  Cashew nut, ground nut kept in room : _0_ No _1_ Yes _9_ NSP  Curdled milk kept in room : _0_ No _1_ Yes _9_ NSP  Dried leaves (mint, quinquiliba, baobab, etc.) : _0_ No _1_ Yes _9_ NSP  Other aliments kept in room : _0_ No _1_ Yes _9_ NSP  If Others, define :……………………………………………  What is the type of roofing of the rooms where your child lives (today and during the first year of life) ?  Corrugated metal roof today : _0_ No _1_ Yes _9_ NSP  Corrugated metal roof 0-1yr : _0_ No _1_ Yes _9_ NSP  Thatched roof today: _0_ No _1_ Yes _9_ NSP  Thatched roof 0-1yr : _0_ No _1_ Yes _9_ NSP  Wooden roof today : _0_ No _1_ Yes _9_ NSP  Wooden roof 0-1yr : _0_ No _1_ Yes _9_ NSP  Cement roof today : _0_ No _1_ Yes _9_ NSP  Cement roof 0-1yr : _0_ No _1_ Yes _9_ NSP  Plaster roof today : _0_ No _1_ Yes _9_ NSP  Plaster roof 0-1yr : _0_ No _1_ Yes _9_ NSP  Other type of roof today : _0_ No _1_ Yes _9_ NSP  Other type of roof 0-1yr : _0_ No _1_ Yes _9_ NSP  If other, define :……………………………………………   1. Which of these objects are in the room where your child sleeps (today and during the first year of life) ?   Mattress in room today : _0_ No _1_ Yes _9_ NSP  Mattress in room 0-1yr : _0_ No _1_ Yes _9_ NSP  Bednet in room today : _0_ No _1_ Yes _9_ NSP  Bednet in room 0-1yr : _0_ No _1_ Yes _9_ NSP  Wardrobe in room today : _0_ No _1_ Yes _9_ NSP  Wardrobe in room 0-1yr : _0_ No _1_ Yes _9_ NSP  Chest, trunk in room today : _0_ No _1_ Yes _9_ NSP  Chest, trunk in room 0-1yr : _0_ No _1_ Yes _9_ NSP  Table in room today : _0_ No _1_ Yes _9_ NSP  Table in room 0-1yr : _0_ No _1_ Yes _9_ NSP  Chair in room today : _0_ No _1_ Yes _9_ NSP  Chair in room 0-1yr : _0_ No _1_ Yes _9_ NSP  Carpet, rug in room today : _0_ No _1_ Yes _9_ NSP  Carpet, rug in room 0-1yr : _0_ No _1_ Yes _9_ NSP  Matting in room today : _0_ No _1_ Yes _9_ NSP  Matting in room 0-1yr : _0_ No _1_ Yes _9_ NSP  Curtains in room today : _0_ No _1_ Yes _9_ NSP  Curtains in room 0-1yr : _0_ No _1_ Yes _9_ NSP  Malagasy fire in room today : _0_ No _1_ Yes _9_ NSP  Malagasy fire in room 0-1yr : _0_ No _1_ Yes _9_ NSP  Other objects in room today : _0_ No _1_ Yes _9_ NSP  Other objects in room 0-1yr : _0_ No _1_ Yes _9_ NSP  If other, define :……………………………………………   1. On what type of bedding does your child sleep (today and during the first year of life) ?   Foam mattress today : _0_ No _1_ Yes _9_ NSP  Foam mattress 0-1yr : _0_ No _1_ Yes _9_ NSP  Plant fibre mattress (straw, etc.) today : _0_ No _1_ Yes _9_ NSP  Plant fibre mattress (straw, etc.) 0-1yr : _0_ No _1_ Yes _9_ NSP  Wool mattress today : _0_ No _1_ Yes _9_ NSP  Wool mattress 0-1yr : _0_ No _1_ Yes _9_ NSP  Feather mattress today : _0_ No _1_ Yes _9_ NSP  Feather mattress 0-1yr : _0_ No _1_ Yes _9_ NSP  Plastic matting today : _0_ No _1_ Yes _9_ NSP  Plastic matting 0-1yr : _0_ No _1_ Yes _9_ NSP  Plant fibre matting (straw, etc.) today : _0_ No _1_ Yes _9_ NSP  Plant fibre matting (straw, etc.) 0-1yr : _0_ No _1_ Yes _9_ NSP  Other type of bedding today : _0_ No _1_ Yes _9_ NSP  Other type of bedding 0-1yr : _0_ No _1_ Yes _9_ NSP  If other, define :……………………………………………   1. Does your child sleep on a pillow ? _0_ No _1_ Yes _9_ NSP   If **No**, go to question **8**  If **Yes**, what type of pillow is it ?  Foam : _0_ No _1_ Yes _9_ NSP  Synthetic  fibres: _0_ No _1_ Yes _9_ NSP  Plant fibres (straw, etc.) : _0_ No _1_ Yes _9_ NSP  Feather : _0_ No _1_ Yes _9_ NSP  Other type of pillow : _0_ No _1_ Yes _9_ NSP  If other, define :……………………………………………   1. Do people smoke in the room where your child lives ?   Today : _0_ No _1_ Yes _9_ NSP  From 0-1yr : _0_ No _1_ Yes _9_ NSP  During the pregnancy of the mother : _0_ No _1_ Yes _9_ NSP   1. What type of heating and lighting are used in the rooms where your child lives ?   Heating and lighting by charcoal : _0_ No _1_ Yes _9_ NSP  Heating and lighting by wood : _0_ No _1_ Yes _9_ NSP  Lighting by candle : _0_ No _1_ Yes _9_ NSP  Lighting by petrol lamp : _0_ No _1_ Yes _9_ NSP  Lighting by flash light : _0_ No _1_ Yes _9_ NSP  Lighting by solar : _0_ No _1_ Yes _9_ NSP  Other types of heating and lighting: _0_ No _1_ Yes _9_ NSP  If other, define :……………………………………………   1. Which of the following products are used or stocked in the rooms where you child lives ?   Insecticide (type Yotox, spirales, etc.) : _0_ No _1_ Yes _9_ NSP  Deodorants (aerosols) : _0_ No _1_ Yes _9_ NSP  Incense : _0_ No _1_ Yes _9_ NSP  Detergents (type Cotol, etc.) : _0_ No _1_ Yes _9_ NSP  Petrol, diesel : _0_ No _1_ Yes _9_ NSP  Other types of products : _0_ No _1_ Yes _9_ NSP  If other, define :……………………………………………  ***Diet :***   1. Has your child had **diarrhoea without fever** or abdominal pains (colic)   **following introduction of non-maternal milk** in his diet (cow or goat’s milk, milk powder) : _0_ No _1_ Yes _9_ NSP  **after a few months** of consuming **non-maternal** (cow or goat’s milk, milk powder) : _0_ No _1_ Yes _9_ NSP   1. Currently, how many times, on average, does your child eat the following aliments ?   *The consumption of certain aliments is seasonal.*  Meat : _1_ Never _2_ <1times/week _3_ 1-2 times/week _4_ ≥1times/day  Fish : _1_ Never _2_ <1times/week _3_ 1-2 times/week _4_ ≥1times/day  Egg : _1_ Never _2_ <1times/week _3_ 1-2 times/week _4_ ≥1times/day  Milk (liquid, powder, curdled) : _1_ Never _2_ <1times/week _3_ 1-2 times/week _4_ ≥1times/day  Banana : _1_ Never _2_ <1times/week _3_ 1-2 times/week _4_ ≥1times/day  Mango : _1_ Never _2_ <1times/week _3_ 1-2 times/week _4_ ≥1times/day  Melon : _1_ Never _2_ <1times/week _3_ 1-2 times/week _4_ ≥1times/day  Orange, lime : _1_ Never _2_ <1times/week _3_ 1-2 times/week _4_ ≥1times/day  Potatoes, sweet potatoes : _1_ Never _2_ <1times/week _3_ 1-2 times/week _4_ ≥1times/day  Vegetables : _1_ Never _2_ <1times/week _3_ 1-2 times/week _4_ ≥1times/day  Millet : _1_ Never _2_ <1times/week _3_ 1-2 times/week _4_ ≥1times/day  Sorghum : _1_ Never _2_ <1times/week _3_ 1-2 times/week _4_ ≥1times/day  Maize : _1_ Never _2_ <1times/week _3_ 1-2 times/week _4_ ≥1times/day  Rice : _1_ Never _2_ <1times/week _3_ 1-2 times/week _4_ ≥1times/day  Wheat (bread, pasta) : _1_ Never _2_ <1times/week _3_ 1-2 times/week _4_ ≥1times/day  Nuts (Cashew, ground nut) : _1_ Never _2_ <1times/week _3_ 1-2 times/week _4_ ≥1times/day  Prawns, dried oysters : _1_ Never _2_ <1times/week _3_ 1-2 times/week _4_ ≥1times/day  Flavouring cubes Maggi : _1_ Never _2_ <1times/week _3_ 1-2 times/week _4_ ≥1times/day  Other : _1_ Never _2_ <1times/week _3_ 1-2 times/week _4_ ≥1times/day  If other, define :……………………………………………  **HISTORICAL SYMPTOMATOLOGY OF ALLERGIC REACTIONS**  ***Asthma :***   1. Has a doctor or nurse **already** said that you child has asthma ?   0 No _1_ Yes _9_ NSP   1. Has your child already breathed noisily or had whistling in his chest whilst breathing   0 No _1_ Yes _9_ NSP  If **No**, go directly to question **6**   1. During his first two years of life, has your child already breathed noisily or had whistling in his chest whilst breathing ?   0 No _1_ Yes _9_ NSP  If **No**, go directly to question **6**  If **Yes**, how many times (before 2 years of age) ?  _1_1time _2_2times _3_ ≥3times _9_ NSP  Between the last two **ramadans**, has your child already breathed noisily or had whistling in his chest whilst breathing ?  0 No _1_ Yes _9_ NSP  If **No**, go directly to question **5**  If **Yes**, at which moment of the year ?  Rainy season : _0_ No _1_ Yes _9_ NSP  Dry season : _0_ No _1_ Yes _9_ NSP  Harvest time : _0_ No _1_ Yes _9_ NSP  Has the noisy breathing of your child been such that it has prevented him from talking normally?  0 No _1_ Yes _9_ NSP  Has your child already had a rasping cough at night that prevents him from sleeping normally ?  0 No _1_ Yes _9_ NSP  ***Rhinitis and allergic  conjunctivitis;***   1. Has your child **already had** problems of a runny nose, **or** a sensation of a blocked nose, **or** an itchy nose, **or** sneezing, or loss of the sense of smell **for more than a week,** irrespective of the frequency of these episodes? _0_ No _1_ Yes _9_ NSP 2. Has your child **already had** problems of a runny nose, **or** a sensation of a blocked nose, **or** an itchy nose, **or** sneezing, or loss of the sense of smell **more than 5 times in one year,** irrespective of the frequency of these episodes? _0_ No _1_ Yes _9_ NSP   Between the **last two ramadans**, has your child **already had** problems of a runny nose, **or** a sensation of a blocked nose, **or** an itchy nose, **or** sneezing, or loss of the sense of smell ?  0 No _1_ Yes _9_ NSP  If **No**, go to question **4**  If **Yes**, at what moment of the year ?  Rainy season : _0_ No _1_ Yes _9_ NSP  Dry season : _0_ No _1_ Yes _9_ NSP  Harvest time : _0_ No _1_ Yes _9_ NSP   1. Has your child **already had** watery eyes, **or** itchy eyes, **or** an allergic limbo-conjonctivitis?   0 No _1_ Yes _9_ NSP  If **No**, go directly to question **1** in the section **Eczema**  Has your child had, between the **last two** **ramadans**, watery eyes, **or** itchy eyes, **or** an allergic limbo-conjonctivitis?  0 No _1_ Yes _9_ NSP  If **No**, go directly to question **5**  If **Yes**, at what moment of the year ?  Rainy season : _0_ No _1_ Yes _9_ NSP  Dry season : _0_ No _1_ Yes _9_ NSP  Harvest time  : _0_ No _1_ Yes _9_ NSP  ***Eczéma ;***  Has your child **already had** skin problems with dry patches or seeping cracked patches and itching ?  0 No _1_ Yes _9_ NSP  If **No**, **the questionnaire has finished.**  Between the **last two** **ramadans**, has your child had skin problems with dry patches or seeping cracked patches and itching ??  0 No _1_ Yes _9_ NSP  If **No**, go directly to question **3**  If **Yes**, at what moment of the year ?  Rainy season : _0_ No _1_ Yes _9_ NSP  Dry season : _0_ No _1_ Yes _9_ NSP  Harvest time : _0_ No _1_ Yes _9_ NSP   1. Have these skin problems affected different parts of the body of your child ?   Scalp : _0_ No _1_ Yes _9_ NSP  Face : _0_ No _1_ Yes _9_ NSP  Around the eyes and ears : _0_ No _1_ Yes _9_ NSP  Armpits : _0_ No _1_ Yes _9_ NSP  Elbow : _0_ No _1_ Yes _9_ NSP  Hands : _0_ No _1_ Yes _9_ NSP  Under the buttocks: _0_ No _1_ Yes _9_ NSP  Groin : _0_ No _1_ Yes _9_ NSP  Behind the knee : _0_ No _1_ Yes _9_ NSP  Feet : _0_ No _1_ Yes _9_ NSP  Other part of body : _0_ No _1_ Yes _9_ NSP  What age did your child have when these skin problems of dry patches, weeping cracked patches or itching appear for the **first time**?  _1_ < 2yr _2_ 2 - 4 yr _3_ ≥ 5yr  Have your child’s skin problems ever been sufficiently important to prevent him from sleeping correctly or waking him up during the night ?  0 No _1_ Yes _9_ NSP  ***Comments :*** Note with reference to which questions these comments apply  …………………………………………………………………………………………………………  ……………………………………………………………………………………………………………………  ……………………………………………………………………………………………………………………  ……………………………………………………………………………………………………………………  ……………………………………………………………………………………………………………………  ……………………………………………………………………………………………………………………  ……………………………………………………………………………………………………………………  ……………………………………………………………………………………………………………………  ……………………………………………………………………………………………………………………  ……………………………………………………………………………………………………………………  …………………………………………………………………………………………………………………… | |__| AGEBREAST  |__| MALAR  |__| TUBTRT  |__| HEMINTH  |__| AMOEBA  |__| MEASLES  |__| VACFJ  |__| VACHEPB  |__| VACMEASLE  |__| VACMUMPS  |__| VACRUBEL  |__| VACTUB  |__| VACDTCP  |__| VACTY  |__| VACMENIN  |__| VACHIB  |__| DOGTODAY  |__| DOG01YR  |__| CATTODAY  |__| CAT01YR  |__| SHEEPTODAY  |__| SHEEP01YR  |__| GOATODAY  |__| GOA01YR  |__| CHICTODAY  |__| CHIC01YR  |__| RODTODAY  |__| ROD01YR  |__| COCTODAY  |__| COC01YR  |__| OTHTODAY  |__| OTH01YR  ……………………………… NAMEOTH  |__| CDOGTODAY  |__| CDOG01YR  |__| CCATODAY  |__| CCAT01YR  |__| CSHEEPTODAY  |__| CSHEEP01YR  |__| CGOATODAY  |__| CGOA01YR  |__| CCHICTODAY  |__| CCHIC01YR  |__| CHORSTODAY  |__| CHORS01YR  |__| CCOWTODAY  |__| CCOW01YR  |__| CRODTODAY  |__| CROD01YR  |__| COTHTODAY  |__| COTH01YR  ………………………………...NAMEOTHC  |__| MIL  |__| SORG  |__| MAIZ  |__| RICE  |__| WHEA  |__| BISCUI  |__| MANIOC  |__| NUTP  |__| MILKCURD  |__| LEAF  |__| OTHALIM  ……………………………… NAMEOTHAL  |__| RMETTODAY  |__| RMET01YR  |__| RTHATDAY  |__| RTHAT01YR  |__| RWOOTODAY  |__| RWOO01YR  |__| RCEMTODAY  |__| RCEM01YR  |__| RPLATODAY  |__| RPLA01YR  |__| ROTHTODAY  |__| ROTH01YR  …………………… NAMEOTHR  |__| MATRTODAY  |__| MATR01YR  |__| BEDNTODAY  |__| BEDN01YR  |__| WARDTODAY  |__| WARD01YR  |__| CHESTODAY  |__| CHES01YR  |__| TABPTODAY  |__| TABP01YR  |__| CHPTODAY  |__| CHA01YR  |__| CARPTODAY  |__| CARP01YR  |__| MATPTODAY  |__| MATP01YR  |__| CURTTODAY  |__| CURT01YR  |__| FIRTODAY  |__| FIR01YR  |__| OTHOBTODAY  |__| OTHOB01YR  ……………………………… NAMEOTHOB  |__| FMATRTODAY  |__| FMATR01YR  |__| PLFMATRTODAY  |__| PLFMATR01YR  |__| WOMATRTODAY  |__| WOMATR01YR  |__| FEATHMTODAY  |__| FEATHM01YR  |__| PLMATTODAY  |__| PLMAT01YR  |__| PLFMATTODAY  |__| PLFMAT01YR  |__| OTHBEDTODAY  |__| OTHBED01YR  ……………………………… NOMAUTLI  |__| PILLOW  |__| PILLF  |__| PILLSYN  |__| PILLPLF  |__| PILLFEATH  |__| OTHPILL  ……………………………… NAMEOTHPILL  |__| SMOKTODAY  |__| SMOK01YR  |__| SMOKPREG  |__| CHELCHAR  |__| CHELWOO  |__| LCAND  |__| LLAMP  |__| LTORCH  |__| LSOLAR  |__| OTHHEL  ……………………………… NAMEOTHHEL  |__| INSECTIC  |__| DEODORA  |__| INCENSE  |__| DETERGEN  |__| PETROL  |__| OTHPROD  ……………………………… NAMEOTHPR  |__| DIARINT  |__| DIARMONTH  |__| CONSMEAT  |__| CONSFISH  |__| CONSEGG  |__| CONSMILK  |__| CONSBANA  |__| CONSMANG  |__| CONSMELON  |__| CONSORAN  |__| CONSPOT  |__| CONSVEG  |__| CONSMIL  |__| CONSSORG  |__| CONSMAIS  |__| CONSRICE  |__| CONSWHEA  |__| CONSNUT  |__| CONSPRAWN  |__| CONSCUBE  |__| OTHALCON  ……………………………… NAMEOTHAL  |__| ASTHMA  |__| WHISTLING  |__| WHISTL2YR  |__| NBWHIS2YR  |__| WHISTL2RA  |__| WHISTLRS  |__| WHISTLDS  |__| WHISTLHT  |__| PREVTALK  |__| TOUSECHE  |__| RHIN1WEEK  |__| RHIN5FAN  |__| RHIN2RAM  |__| RHINRS  |__| RHINDS  |__| RHINHT  |__| CONJALER  |__| CONJ2RAM  |__| CONJRS  |__| CONJDS  |__| CONJHT  |__| ECZEMA  |__| ECZE2RAM  |__| ECZEMARS  |__| ECZEMADS  |__| ECZEMAHT  |__| ECZESCALP  |__| ECZEFAC  |__| ECZEEYEEAR  |__| ECZEARMPIT  |__| ECZEELBOW  |__| ECZEHAND  |__| ECZEBUTT  |__| ECZEGROIN  |__| ECZEKNEE  |__| ECZEFEET  |__| ECZEOTH  |__| AGECZEMA  |__| IRRECZEM |
