# Supplementary material for: Risk factors associated with asthma, atopic dermatitis and rhinoconjunctivitis in a rural Senegalese cohort
Source: Allergy Asthma Clin Immunol. 2015 Aug 25;11(1):24. doi: 10.1186/s13223-015-0090-0 (PMC4547418; doi:10.1186/s13223-015-0090-0)
Supplement: Additional file 2. — Allergy classification criteria, description of ISAAC variables and details of allergens and immunoglobulin E titres [file 13223_2015_90_MOESM2_ESM.doc]

**1. Allergy classification criteria**

*Asthma* – *severe* symptoms if the child had “wheezing or whistling in the chest before the age of two years” and “more than three times” *or* severe enough to “limit his/her speech”; *moderate* symptoms if the child had “wheezing or whistling in the chest before the age of two years” and “in the past 12 months”; and *none* otherwise.

*Allergic rhinoconjunctivitis* – *severe* symptoms if the child had “sneezing, runny or stuffy nose in the past 12 months” and “more than five times a year”, and “itchy, watery eyes or tropical endemic limboconjunctivitis (TELC) in the past 12 months”; *moderate* symptoms if the child had “sneezing, runny or stuffy nose in the past 12 months”, and “itchy, watery eyes or TELC in the past 12 months”; and *none* otherwise.

*Atopic dermatitis* – *severe* symptoms if the child had “scaly or exudating, crusted and pruritic patches in the past 12 months” and “affecting any of the following characteristic areas: face, around the ears or eyes, folds of armpits or elbows or groin, behind the knees, under the buttocks”, and “onset of symptoms before the age of two years”; *moderate* symptoms if the child had “scaly or exudating, crusted and pruritic patches in the past 12 months” and “affecting any of characteristic areas (see above)”, and “onset of symptoms before the age of four years”; and *none* otherwise.

**2. Immunoglobulin E titres**

Specific IgE titres against mosquito spp. salivary gland extract, house dust mite spp. and a mix of pollen allergens were measured by ELISA. A panel of allergens of potential pertinence to the three classes of allergy was used: (i) Salivary gland extracts (SGE) of three mosquito species present in the study cohorts, *Aedes aegypti*, *Anopheles gambiae sensu stricto*, and *Culex quinquefasciatus* as described; (ii) House dust mite spp. *Dermatophagoides farinae* and *Dermatophagoides pteronyssinus*; (iii) a mix of pollen allergens from five ubiquitous Poaceae spp. [Cock’s-foot (*Dactylis glomerata*), Timothy grass (*Phleum pratense*), Sweet Vernal grass (*Anthoxanthum odoratum*), Perennial ryegrass (*Lolium perenne*), Kentucky Bluegrass (*Poa pratensis*)] (all from Stallergenes, France).

**Supplementary Figure. Specific IgE levels against house dust mite spp.** extracts. Shown are means (±SEM).

**3. Summary of ISAAC variables**

1. Intrinsic features: age, sex, body weight (kg), height (cm), brachial perimeter (mm), height-for-age and categorised into low growth rate (if <-2), birth weight, Body Mass Index, Body Mass Index-for-age and categorised into two new factors, underweight (moderate if BMI-age <-2 and severe if BMI-age <-3) and overweight (overweight if BMI-age >1, obese if BMI-age >2), weight-for-age and classified into underweight (if <-2) and state of malnutrition.

2. History of pathogen infection and vaccination status: infection with helminths, malaria, measles, tuberculosis, amoeba. Vaccination for Yellow fever, Tuberculosis, Measles, Hepatitis B, Haemophilus influenza type B, DCTP.

3. Current infestation with worms

4. House/Room environment (furniture etc): Number of objects in room (2-11), table, cupboard, chest of drawers, curtain, carpet/rug, mat, chest, insecticide treated bednet, mattress, synthetic bedding (exclusively so or not), plant fibre bedding (e.g. straw and dry grass etc in the mattress, exclusively so or not), animal product bedding (feathers etc, exclusively or not), pillow, thatched roof, corrugated iron roof, urn.

5. House/Room environment (chemical): Incense burner, Malagasy portable iron oven, gas bottles, charcoal, candle, petrol lamp, electric batteries, solar panels, pesticide, deodorant, detergent, petrol/diesel, butane bottles, fertilizer, current tobacco smoking, smoking during pregnancy.

6. Animals and siblings: contact at least once a week with dog, cat, chicken, goat, sheep, reptile, rodent, donkey, cow; presence in room of dog, cat, chicken, goat, sheep, reptile, rodent, donkey, cow, bat, cockroach. Also considered was the impact of the presence of older siblings (<15 years old) as either a continuous (number of older siblings) or dichotomous variable (yes/no presence of older siblings).

7. Storage of food products: millet, wheat, sorghum, rice, maize, manioc, couscous, nuts, curdled milk, whey, other milk products, dried leaves.

8. Consumption: age stopped exclusive breast feeding, age of weaning, diarrhoea without fever following introduction of non-maternal milk (no, yes, not weaned), diarrhoea without fever after several months following introduction of non-maternal milk (no, yes, not weaned), frequency of consumption of the following food products (never, less than once/week, 1-2 times/week, at least once a day): millet, wheat, sorghum, rice, maize, potatoes, meat, fish, seafood (prawns, mangrove oysters, *Cymbium* spp. molluscs), eggs, milk, bananas, oranges, water melon, mango, nuts, vegetables, cooking cubes, other.
